# Smart Pneumatic Artificial Muscle Using a Bend Sensor like a Human Muscle with a Muscle Spindle

**DOI:** 10.3390/s22228975

**Published:** 2022-11-19

**Authors:** Norihiko Saga, Kunio Shimada, Douhaku Inamori, Naoki Saito, Toshiyuki Satoh, Jun-ya Nagase

**Affiliations:** 1 School of Engineering, Kwansei Gakuin University, 1 Gakuenuegahara, Sanda 669-1330, Japan; 2 Faculty of Symbiotic Systems Sciences, Fukushima University, 1 Kanayagawa, Fukushima 960-1296, Japan; 3 Engineering Department, Strain Gage Engineering Section, Minebeamitsumi Inc., 1-1-1, Katase, Fujisawa 251-8531, Japan; 4 Department of Intelligent Mechatronics, Akita Prefectural University, 84-4Aza-Ebinokuchi, Yurihonjo 015-0055, Japan; 5 Faculty of Advanced Science and Technology, Ryukoku University, 1-5 Yokotani, Seta Oe-cho, Otsu 520-2194, Japan

**Keywords:** artificial muscle, pneumatic actuator, strain gage, bend sensor, muscle spindle

## Abstract

Shortage of labor and increased work of young people are causing problems in terms of care and welfare of a growing proportion of elderly people. This is a looming social problem because people of advanced ages are increasing. Necessary in the fields of care and welfare, pneumatic artificial muscles in actuators of robots are being examined. Pneumatic artificial muscles have a high output per unit of weight, and they are soft, similarly to human muscles. However, in previous research of robots using pneumatic artificial muscles, rigid sensors were often installed at joints and other locations due to the robots’ structures. Therefore, we developed a smart actuator that integrates a bending sensor that functions as a human muscle spindle; it can be externally attached to the pneumatic artificial muscle. This paper reports a smart artificial muscle actuator that can sense contraction, which can be applied to developed self-monitoring and robot posture control.

## 1. Introduction

In terms of care and welfare activities, shortage of young people’s labor and increase in their work are presenting important social problems regarding an increase in low birth rates and longevity. It is therefore anticipated that robots will perform rehabilitation and daily domestic tasks for nursing care and welfare services. These robots must be flexible and safe in their contact with humans [1,2,3].

A pneumatic artificial muscle (PAM) can satisfy this demand. It has a high power-to-weight ratio. Moreover, it has a low cost and a compact, flexible structure. Therefore, this actuator is anticipated for use in a mechanical system used in circumstances that demand contact with people. PAMs are classifiable into several types based on their structures and drive mechanisms. In 1961, Joseph McKibben developed the PAM, which was designated as a McKibben-type PAM [4,5,6]. Numerous studies of its arrangement and examinations of its control efficiency were conducted to underscore possible applications in robot technology [7,8,9]. However, it lacked durability because the nylon fibers placed around the rubber would rub and break during driving. Moreover, highly compressed air was necessary to drive it [10,11,12].

To mitigate these faults, a pneumatic rubber artificial muscle, in which high-intensity carbon fiber was built into the silicone tube, was developed. This actuator has reinforced fibers arranged longitudinally. Therefore, this PAM has high durability and a high shrinkage ratio as compared with the McKibben-type PAM.

In past studies of robots using PAMs, hard sensors such as encoders [13,14], potentiometers [15] and Hall effect sensors [16,17] have been most used. Therefore, no matter how flexible the construction of a robot might have been, each joint itself was highly rigid. Consequently, the problem of safety persisted.

A sensory organ called the muscle spindle senses muscular contraction, as does the Golgi tendon organ in the skeletal muscle. It has a self-defense function to avoid excessive muscular extension. Examples of soft sensors that can be integrated into PAMs include three-dimensional helical sensors [18,19] that replace reinforcing fibers with conductive fibers [20]; conductive wires [21,22] as sensors; etc. However, the manufacturing process is complex and requires integration into the actuator through a three-dimensional manufacturing process. The cost of the actuator itself is high.

We focused on the fact that there was a correlation between the amount of circumferential deformation and the amount of axial contraction of PAMs during operation. Therefore, we developed a smart PAM system that estimates the amount of contraction of a PAM based on the amount of circumferential deformation by means of a flexible flexure sensor based on strain gauges attached to the surface of the PAM. Its self-monitoring function enables detection of any excessive contraction and deterioration. This paper proposes a smart PAM system that resembles human muscle through use of muscle spindles, and introduces its structure, manufacturing method and sensing technique to explain its effectiveness.

## 2. Pneumatic Artificial Muscle Reinforced by Carbon Fibers

### 2.1. Simplified Manufacturing Method and Detailed Configuration

**Figure 1:**
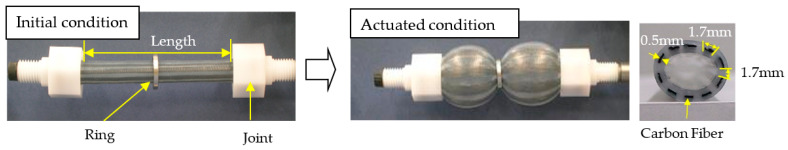
Photos of PAM.

**Figure 2:**
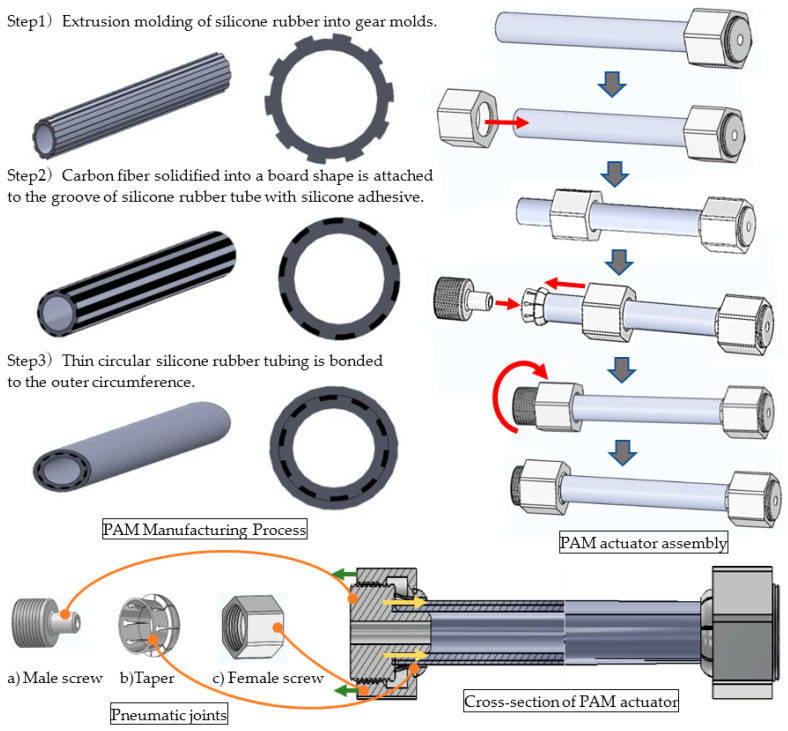
Structure of PAM.

Figure 1 portrays a photograph of the PAM in its actuated condition. Table 1 shows the specifications of the PAM used for this study. In addition, Figure 2 portrays the structures of a McKibben-type PAM and a PAM reinforced by straight fibers, the latter of which was the PAM type used in this study. For manufacture of the PAM, the groove of the silicon tube, extruded into a gear type as shown in the figure, was a bonded carbon fiber strip, further covering the thin-film silicon tube of the cylinder on the outer periphery. In addition, the configuration of the pneumatic joint section for the actuator, as shown in the figure, consisted of a male threaded portion, a female threaded portion and a tapered portion. The female threaded portion passed through the actuator in advance; then a tapered portion was mounted on the outer periphery of the end portion of the actuator and finally, a male threaded portion was inserted into the actuator. As the screw was tightened, the tapered portion of the silicone rubber tube was restrained from the periphery to prevent air leakage. The tapered portion had slits to accommodate slight changes in the PAM’s thickness, outer diameter and inner diameter.

### 2.2. Fundamental Characteristics

**Figure 3:**
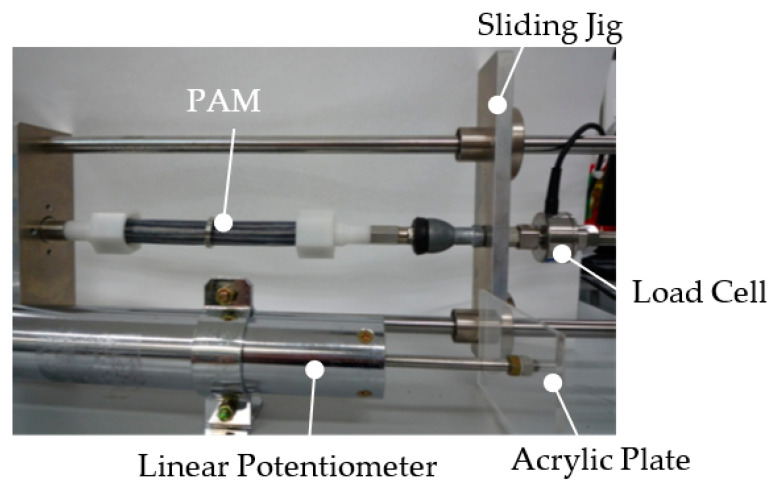
Experimental system.

**Figure 4:**
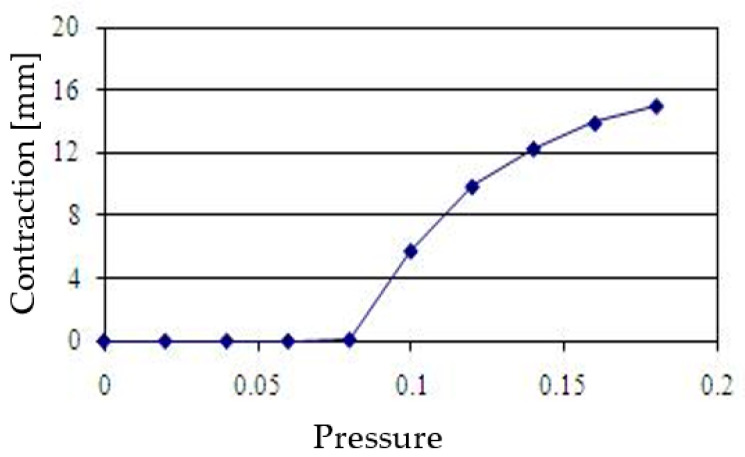
Relationship between pressure and contraction.

Figure 3 shows the experimental setup for measuring the relationship between input pressure and contraction. In this experiment, the input air pressure was set from 0 MPa to 0.18 MPa, in 0.02 MPa increments. One end of the PAM was connected to a fixed plate. The other end was connected to a slider that moved freely in the horizontal direction. The PAM was therefore driven along the slider. The PAM was then extended and retracted by the sliding jig, and the amount of expansion and contraction of the acrylic plate connected to this jig (equal to the amount of expansion and contraction of the PAM) was measured with a linear potentiometer (DLT-100AS: KYOWA ELECTRONIC INSTRUMENTS Co., Ltd., Tokyo, Japan). Compressed air input to the PAM was provided by an air compressor (YC-4: Yaesaki Kuatsu Co., Ltd., Itami, Japan). Air pressure input to the PAM was controlled by an electro-pneumatic proportional valve (ETR-200-1; Koganei Seisakusho, Tokyo, Japan). Accuracy of the linear potentiometer used demonstrated a ±100 mm rated capacity and nonlinearity within ±0.5%; 100 mm × 0.5% = ±0.5 mm. Figure 4 shows the characteristics of the relationship between input pressure and the amount of contraction. Our PAM had a dead zone between 0 MPa and 0.08 MPa, contracted significantly at 0.08~0.12 MPa and increased slowly afterward.

## 3. Strain-Gage-Type Bend Sensor

### 3.1. Circuit

**Figure 5:**
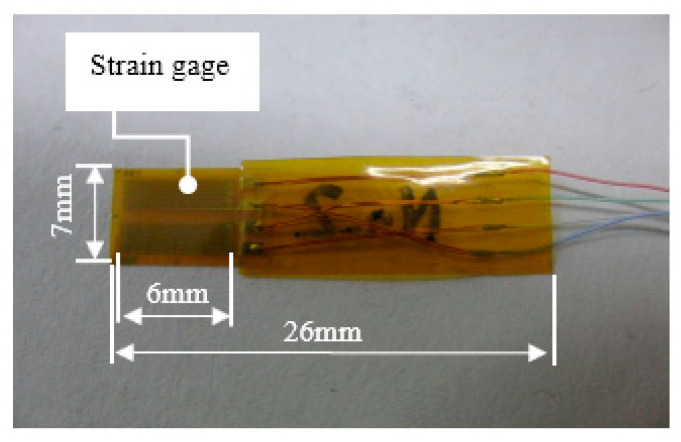
Bend sensor.

A photograph of our developed bend sensor is presented in Figure 5; “bend” denotes changing curvature of an object in this paper. The base plate thickness of this sensor was 50 μm; it consisted of an organic molecular film and a resin film that included a polyimide membrane. If the test piece of the bend sensor were a material that was difficult to adhere and axial strain were greater than some dozens of percent, then it could have easily become unglued or impossible to measure (the strain would have exceeded the strain limit if it did not become unglued).

**Figure 6:**
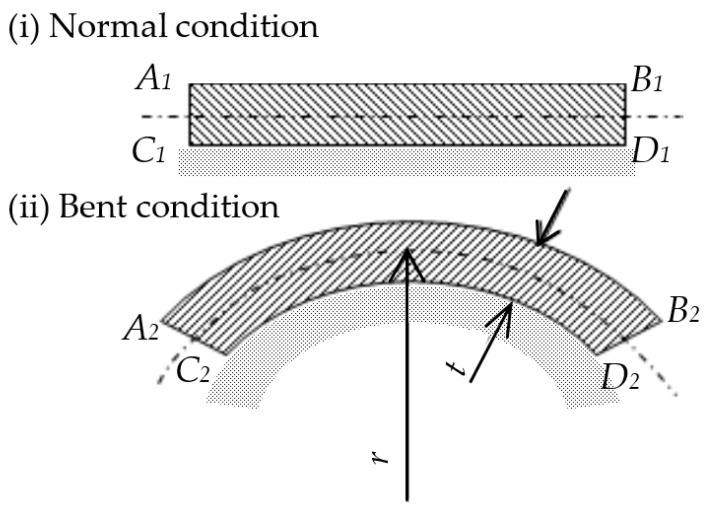
Principle of bend sensor.

A strain-gage-type bend sensor was therefore proposed as a sensor that could measure the bend without adhering. As shown in Figure 6, when the sensor bent, the length of *A*_1_*B*_1_ = *C*_1_*D*_1_ changed to *A*_2_*B*_2_, *C*_2_*D*_2_, and the relation of each length became *A*_2_*B*_2_ > *A*_1_*B*_1_, *C*_2_*D*_2_ > *C*_1_*D*_1_. The following equations hold:

(1) $$\varepsilon_{1}=\frac{A_{2}B_{2}-A_{1}B_{1}}{A_{1}B_{1}}=\frac{2\pi (r+t/2)-2\pi r}{2\pi r}$$

(2) $$\varepsilon_{2}=\frac{C_{2}D_{2}-C_{1}D_{1}}{C_{1}D_{1}}=\frac{2\pi (r-t/2)-2\pi r}{2\pi r}$$

**Figure 7:**
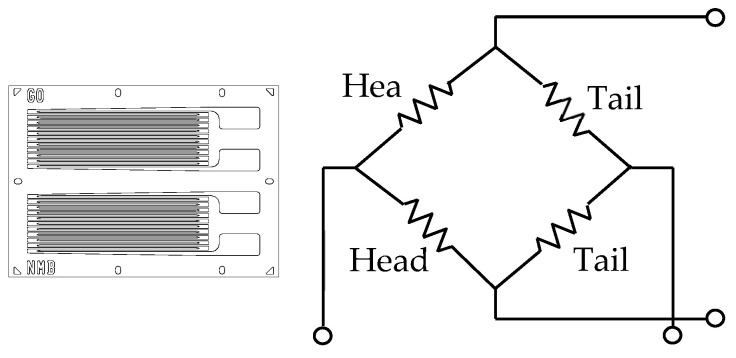
Configuration and circuit diagram.

Herein, strain of the upper side is ε_1_, that of the lower side is ε_2_ and thickness of sides A_1_B_2_C_3_D_4_ is t. This equation indicates that strain increases with decreased curvature. This bend sensor had a bridge circuit with a four-active-gage method: two strain gages were arranged in A_1_B_1_ and two were arranged in C_1_D_1_. The strain gages were adhered to each other, side by side. The construction and bridge circuit are portrayed in Figure 7.

To secure fatigue life without exceeding the strain’s limit of the strain gage, even if the curvature were small, it was necessary for thickness (t) to be designed to be as thin as possible. This bend sensor had no stress in the adhesion layer because the boundary between two strain gages that were adhered to each other was the center of thickness (t). Moreover, this sensor had no conventional test body. It was designed to be thin and was not adhered to the object to be measured. Therefore, few obstructions hindered bending of the measured object, and bending could be measured irrespectively of surface extension.

### 3.2. Calibration

**Figure 8:**
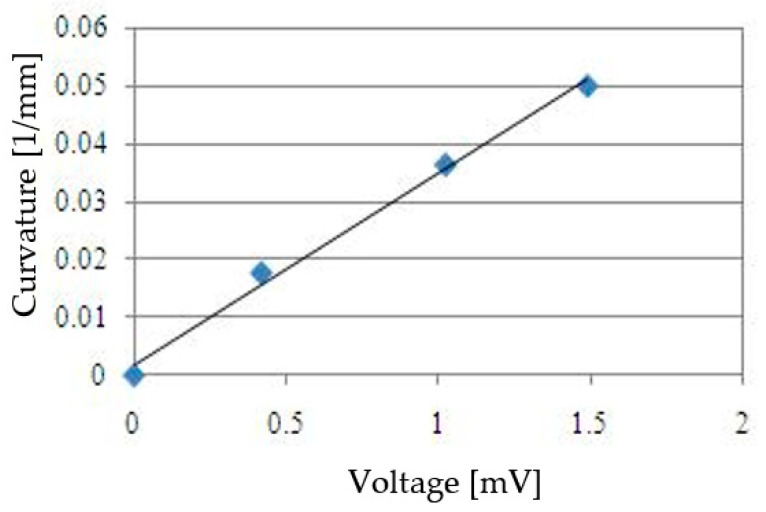
Relationship between curvature and voltage.

To calibrate the bend sensor, the relation between curvature and output voltage was examined through experimentation. The surface of the bend sensor was glued to that of the cylindrical body. Then, curvature and output voltage were measured. Three cylindrical bodies, with respective diameters of 20 mm, 27.5 mm and 56.5 mm, were used. Figure 8 shows the experimental results. Each curvature presented in Figure 8 is the average of three measured data. These results show that output voltage was related to curvature. The equation of curvature (*C*) for output voltage (*V*) is shown below.

(3) C=0.0332V+0.0017

## 4. Estimation Method of Contraction from Curvature

**Figure 9:**
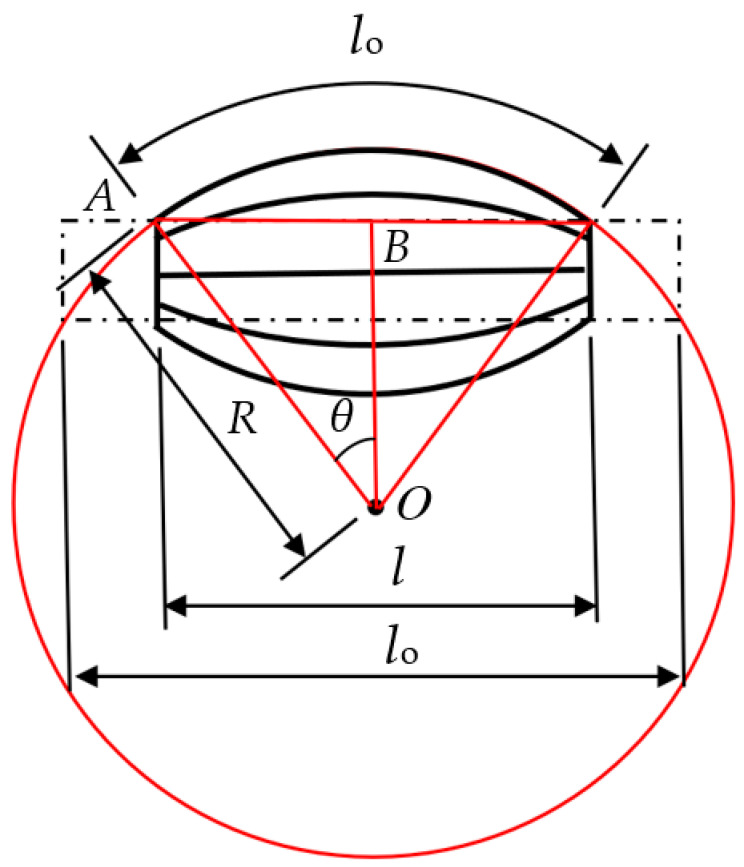
Geometry of PAM.

This section describes the method of estimating PAM contraction from curvature measured by the bend sensor. The sign and the basic specifications used in this section are shown below. The tube curvature when a PAM contracts, *l*_0_, is regarded as the arc of a circle, as shown in Figure 9.

Therein, the endpoint of one side of the tube of the PAM is defined as A, and the center of the circle is O. When the two ends of the tube of the PAM are connected, the point of intersection with a perpendicular line down from O is defined as B. When the angle between AO and OB is defined as *θ*, it is derived geometrically as

(4) $$\theta =\frac{l_{0}}{4\pi R}\times 360$$

where *R* is the curvature radius that is the inverse of the curvature (ρ), and *l*_0_ is length in the axis direction of the tube before contraction of the PAM. Length in axis direction of the tube after contraction is defined as *l*, which is given as shown below:

(5) l=2Rsinθ

From Equations (4) and (5), PAM contraction (*S*) is expressed as follows:

(6) $$S=l_{0}-l=l_{0}-2R\sin\left(\frac{l_{0}}{2R\pi }\times 360\right)$$

## 5. Estimation Method of Contraction from the Curvature

### 5.1. Mounting the Bend Sensor to a PAM

**Figure 10:**
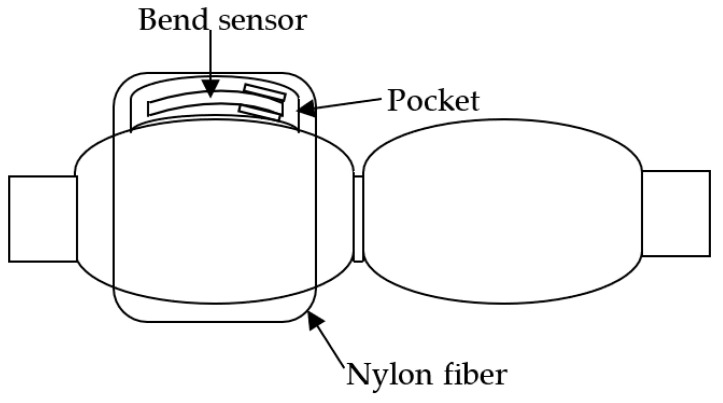
View of PAM with nylon fiber.

PAM contraction is prevented when a bend sensor is bonded to a PAM as a general strain gage [12]. Therefore, a flexion sensor was attached to a part of the circular ring of highly elastic nylon fiber used for stockings, with a pocket for sensor insertion made of the same material. This ensured positioning of the sensor as well as slippage between the flexion sensor and the PAM, thereby slightly preventing contraction of the PAM. A structural drawing of the PAM, with the bend sensor in the pocket made of nylon fiber, is presented in Figure 10.

### 5.2. Evaluation

**Figure 11:**
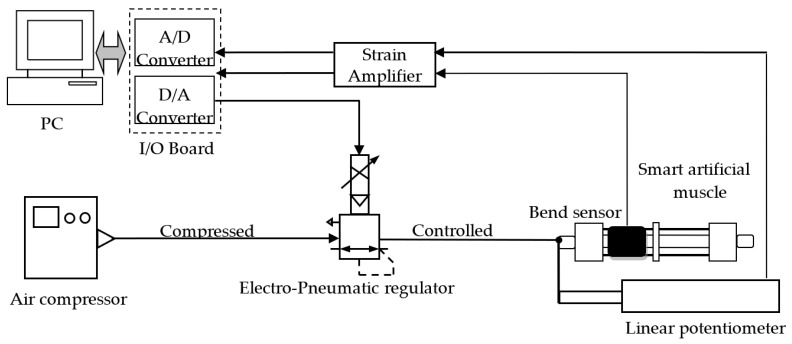
Control system of smart PAM.

**Figure 12:**
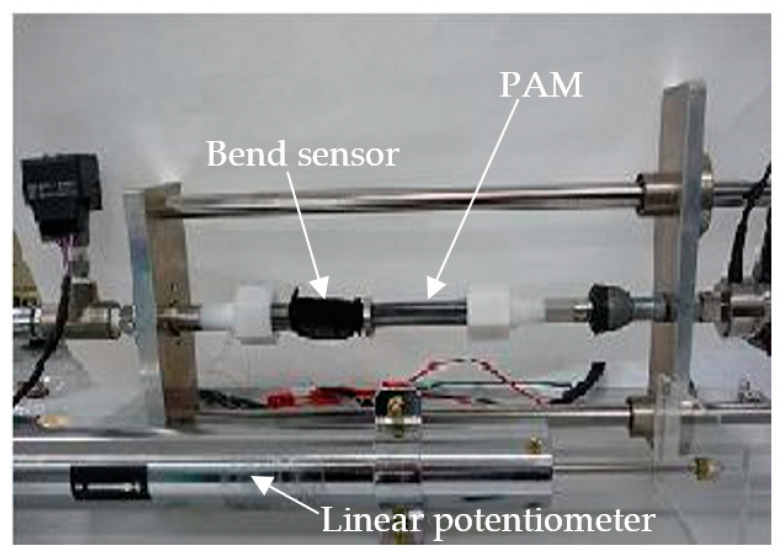
Experimental setup.

Figure 11 shows the smart PAM control system. Figure 12 is a photograph of the experimental system. The experimental procedure was the same as in Section 2.2. The bending sensor was mounted as shown in Figure 12.

**Figure 13:**
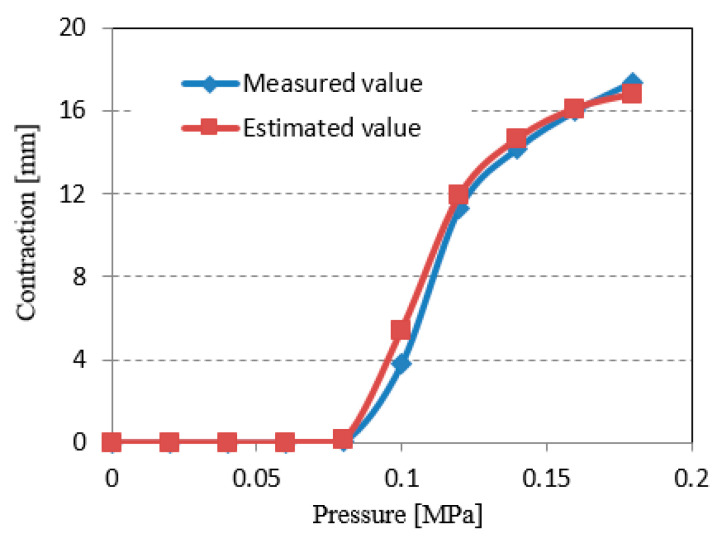
Comparison of actual measured value and estimated value using bend sensor.

In this evaluation, estimated contraction, calculated based on curvature of the tube that was measured using the bend sensor when the PAM contracted, was compared with actual contraction measured by a linear potentiometer (DLT-100AS; Kyowa Corp., Tokyo, Japan). These measured data were input into the PC through a strain amplifier (MULTZ-ACE 6G01, NEC Avio, Tokyo, Japan). Figure 13 shows one example of the experimental results, in which estimated contraction and actual measured contraction are depicted. The experiments for five samples were measured 10 times each. From Figure 13, we can see that the estimated value was almost equivalent to the experimental value at each input pressure, and error was within ±5% in all experiments.

## 6. Conclusions

In this study, we developed a smart PAM system consisting of a strain-gage-type bend sensor and a PAM reinforced by straight fibers. We also verified its effectiveness by comparing estimated contraction, calculated based on curvature that was measured from the developed bend sensor, with actual measured contraction. Our conclusions are the following:

(1) The developed bend sensor can be implemented easily in a PAM. Furthermore, a smart PAM system using the bend sensor can estimate PAM contraction based on curvature measured by the flat flexible bending sensor. The contraction can be measured without prevention of contraction of the muscle because the bending sensor is not bonded to the PAM and is allowed to slip due to bending deformation.
(2) Estimated contraction, as calculated based on curvature measured by the bend sensor, was compared with actual measured contraction. Estimated contraction was close to actual measured contraction at all input air pressures. These results indicate that the proposed smart PAM system can detect excessive contraction in the system and prevent injury to the muscle.

## Figures and Tables

**Table 1:** Specifications of PAM.

Length	100 mm
Outer Diameter	12 mm
Thickness	2 mm
Weight (With Ring and Joint Part)	38 g
Pneumatic Joints	POM
Type of Rubber	Silicon Rubber
Type of Fiber	Carbon Fiber
Number of Fiber Bundles	10
